# Gene Expression Patterns of Royan Human Embryonic Stem Cells Correlate with Their Propensity and Culture Systems 

**DOI:** 10.22074/cellj.2019.6128

**Published:** 2019-06-15

**Authors:** Hassan Rassouli, Mona Khalaj, Seyedeh-Nafiseh Hassani, Shiva Nemati, Ghasem Hosseini Salekdeh, Hossein Baharvand

**Affiliations:** 1 Department of Molecular Systems Biology, Cell Science Research Center, Royan Institute for Stem Cell Biology and Technology, ACECR, Tehran, Iran; 2 Department of Stem Cells and Developmental Biology, Cell Science Research Center, Royan Institute for Stem Cell Biology and Technology, ACECR, Tehran, Iran; 3 Department of Systems Biology, Agricultural Biotechnology Research Institute of Iran, Agricultural Research, Education, and Extension Organization, Karaj, Iran; 4 Department of Molecular Sciences, Macquarie University, Sydney, NSW, Australia; 5 Department of Developmental Biology, University of Science and Culture, Tehran, Iran

**Keywords:** Differentiation, Gene Expression, Pluripotency, Propensity, Stem Cell

## Abstract

**Objective:**

Human embryonic stem cells (hESCs) have the potential to give rise to all types of cells in the human body when appropriately induced to differentiate. Stem cells can differentiate spontaneously into the three-germ layer derivatives by embryoid bodies (EBs) formation. However, the two-dimensional (2D) adherent culture of hESCs under defined conditions is commonly used for directed differentiation toward a specific type of mature cells. In this study, we aimed to determine the propensity of the Royan hESC lines based on comparison of expression levels of 46 lineage specific markers.

**Materials and Methods:**

In this experimental study, we have compared the expression of lineage-specific markers in hESC lines during EB versus adherent-based spontaneous differentiation. We used quantitative real-time polymerase chain reaction (qRT-PCR) to assess expressions of 46 lineage-specific markers in 4 hESC lines, Royan H1 (RH1), RH2, RH5, and RH6, during spontaneous differentiation in both EB and adherent cultures at 0, 10, and 30 days after initiation of differentiation.

**Results:**

Based on qRT-PCR data analysis, the liver and neuronal markers had higher expression levels in EBs, whereas skin-specific markers expressed at higher levels in the adherent culture. The results showed differential expression patterns of some lineage-specific markers in EBs compared with the adherent cultures.

**Conclusion:**

According to these results, possibly the spontaneous differentiation technique could be a useful method for optimization of culture conditions to differentiate stem cells into specific cell types such ectoderm, neuron, endoderm and hepatocyte. This approach might prove beneficial for further work on maximizing the efficiency of directed differentiation and development of novel differentiation protocols.

## Introduction

Embryonic stem cells (ESCs) divide indefinitely and give rise to all different cell types during differentiation. These unique properties make them invaluable cell sources for a wide range of applications in regenerative medicine, cell therapy, disease modeling, drug screening, gene delivery, and other research. However, despite their potential, human ESCs (hESCs) have not been successfully introduced into the clinic setting. Numerous obstacles must be overcome prior to their efficient use in cell replacement therapies. In addition to ethical issues that concern the embryonic source of hESCs (1), the most important technical challenges that stem cell researchers face are concerns about the safety of the cells (2), their purity (3), host immune system rejection (4), efficiency and reproducibility of differentiation , and cell transduction or post-transplantation issues (5).

Optimization of the current differentiation protocols is a major challenge in stem cell research. The ability to efficiently generate pure populations of mature cell types from stem cells over a reasonable period of time is a major challenge. There are several protocols that enable differentiation of hESCs into diverse cell types like cardiomyocytes (6, 7), dopaminergic neurons (8), definitive endoderm (9), human hepatic competent endoderm (10), neural cells (11), and insulin-producing cells (12). Different approaches vary in terms of chemical and mechanical factors, and include the composition of growth factors and soluble inducers, forces applied in the cultured cells, and the culture system used for differentiation. Modifications in the chemical factors in stem cell media have been intensely analyzed in order to enhance differentiation. However, researchers have recently noted the significant role of physical and mechanical variables on differentiation efficiency (13). Cell-cell and cell-matrix interactions have significant roles in cell fate decisions (14). The combination of chemical and mechanical factors that regulate the stem cell fate in vivo can influence the direction and efficiency of lineage specifications in vitro. Therefore, depending on the mature cell type of interest, it is critical to provide stem cells with the appropriate culture system. 

The following 3 main methods are commonly used for stem cell culture: i. Suspension culture and formation of embryoid bodies (EB), ii. Monolayer culture of stem cells on extracellular matrix proteins, and iii. Culture of ESCs on a feeder layer (15). Some protocols for directed differentiation of ESCs use an adherent culture system (16, 17), whereas others suggest a suspension culture (18-20). Advantages and disadvantages associated with both methods make it difficult to choose one method over the other. Stem cells are cultured in monolayers in the adherent system, and the cells divide and grow in a two-dimensional (2D) manner. The soluble inducing factors should be added to the medium for initiation of differentiation, which would allow the cells to have homogenous access to soluble factors in the medium. However, the 2D culture systems do not provide in vivo interactions like cell-cell and cell-matrix connections. The differentiation procedure in the suspension method is basically different compared to the adherent system. In the suspension culture, ESCs usually form three- dimensional (3D) cellular aggregates that lead to the formation of EBs, which begin to spontaneously differentiate before the addition of any soluble inducers (21). EBs resemble the initial stages of embryonic development, in which they form primary embryonic germ layers (ectoderm, mesoderm, and endoderm) (22). The spatial interactions between cells in EBs help direct cell fate determination through signaling pathways that are mainly absent in adherent cultures (23-25). However, as these EBs increase in size, it is difficult for soluble factors to diffuse into the inner cell layers of the aggregate.

Some of the directed differentiation protocols include an initial step of EB mediated spontaneous differentiation followed by addition of soluble inducing factors to the cell aggregates. The differences in dimensions of the suspension and adherent culture systems result in significant differences between cell- cell and cell-matrix interactions, which highlights the importance of a proper choice of a 2D versus 3D stem cell culture system. Several reports have theoretically compared the adherent and suspension culture systems and their effects on the overall differentiation efficiency of stem cells, but there is no report of an experimental comparison (26, 27). In theory, pros and cons exist for both culture systems, and the choice of the appropriate culture system necessitates experimental tests for detailed examination and comprehensive analysis. 

Different signal transduction pathways play roles in the in vivo differentiation of stem cells into each type of mature cells. The microenvironmental factors that govern a cell’s fate in the body include structural, biochemical, mechanical, physiological, and hydrostatic stimuli (28). The environmental triggers required to induce differentiation toward one lineage may differ completely from other lineages; thus, it is reasonable that the choice of culture system for directed differentiation must be cell type-specific. Some cell types might differentiate more efficiently under 2D conditions, whereas other cell types prefer 3D culture systems. Stem cell researchers are seeking straightforward and accurate experimental methods to provide critical information to maximize the efficiency of differentiation for specific cell types.

EB formation and spontaneous differentiation of ESCs result in heterogeneous populations of differentiated cells that include cells of the primary germ layers as well as some of the mature cell types that they produce. Spontaneous differentiation could be the first step for production of pure populations of desired cell types. The convenience of spontaneously differentiated ESCs makes them a useful research tool to provide researchers with valuable information about stem cell behavior, even during directed differentiation. It has been shown that comparative expression of lineage- specific markers during spontaneous differentiation of ESC lines is a good representative of their relative potentials for directed differentiation toward different lineages (29, 30). In these studies, researchers have analyzed the expression levels of lineage-specific markers at various time points after spontaneous differentiation of several distinct ESC lines. Based on the comparative marker expression levels of each lineage, the researchers could hypothesize which ESC line had a higher intrinsic propensity to differentiate toward a specific lineage or cell type. Directed differentiation was then used to validate their hypothesis. The results have proven that spontaneous differentiation is an informative method. Simplicity and reliable data over a relatively short period of time, as well as the cost effectiveness of spontaneous differentiation, make it an interesting approach to study stem cell behavior during differentiation. Since the neural cells differentiation potencies have great potential for cell therapy and treatment of neurological disorders (31).

In this study, we used spontaneous differentiation to optimize differentiation in 4 Royan hESC lines. These hESC lines were cultured under both adherent and suspension culture conditions and we compared expression levels of 46 lineage specific markers to determine the propensity of each of the Royan hESC lines. Subsequently, direct differentiation for neural cells and hepatocytes was done to confirm the spontaneous differentiation results.

We evaluated our hypothesis by focusing on the expression level of neural stem cells lineage specific markers (in two undifferentiated and differentiated states with adherent or suspension culture system) like NESTIN, SOX1, NEUROD1, NCAM, PAX6, PDGFRa and GFAP (as general neural stem cell markers), and b-Tubulin (neural differentiation marker) (32) in spontaneously differentiated samples and specific neural subtypes markers like TH (marker for dopaminergic neural subtypes) in samples from direct differentiation (33). 

In the other side we evaluated hepatocyte differentiation potency by focusing on spontaneous and direct endodermal layer differentiation and checking the general hepatic lineage specific expression markers like BRACHYURY, GOOSECOID, and SOX17 in two different culturing strategies (adherent and suspension). 

## Materials and Methods

### Human embryonic stem cell culture 

In this experimental study, approved by the Ethical Committee of Royan Institute, we have used 4 hESC lines-Royan H1 (RH1), RH2, RH5, and RH6 (34). RH6 is a male hESC line, whereas the other 3 hESC lines have been derived from female embryos. These hESC lines all have a normal karyotype (35). Passage 15-20 hESCs were transferred to dishes coated with mouse embryonic fibroblasts (MEF) and we derived 3 different biological replicates from each cell line. The hESCs were cultured in standard Dulbecco’s modified Eagle’s media (DMEM, Gibco, USA) complemented 20% knockout serum replacement (KOSR), 2 mM L-glutamine, 0.1 mM ß-mercaptoethanol (BME, Sigma, USA), 100 µg/ml pen/ strep, and 100 ng/ml basic fibroblast growth factor (bFGF, Royan Biotech, Iran) (36).

### Spontaneous differentiation

In order to form EBs, we cultured the hESCs in suspension stem cell medium that contained agarose and fetal bovine serum (FBS, Gibco, USA), without KOSR. The samples were harvested at days 10 and 30 after the initiation of EB formation. For spontaneous differentiation in the adherent settings, we used the same media with slight changes. The cells were cultured on 0.1% gelatin coated plates instead of agarose coated plates.

### RNA extraction and cDNA synthesis

Total RNA was extracted using TRIzol (Sigma, USA) according to the manufacturer’s protocol. The RNA concentration was measured using a Biowave WPA (S 2100) spectrophotometer. We examined the purity of each RNA sample based on 260/280 absorbance ratios. We noted that all samples had a ratio of 1.9-2. The quality of total RNA from all samples was determined by electrophoresis. Ribosomal 50S and 28S bands were sharp and showed approximately 2:1 band intensity, which confirmed that the samples had acceptable RNA integrity and quality.

We used a DNase treatment kit (Fermentas, USA) based on the manufacturer’s instructions for elimination of DNA contamination. Random hexamer primers were used for first strand cDNA synthesis using the Fermentas kit. 

### Quantitative real-time polymerase chain reaction

Totally, we used 48 primers in this study. The sequences of 34 primers were obtained from (29) and 13 primers were designed using Gene Runner and PerlPrimer software (Table S1) (See Supplementary Online Information at www.celljournal.org). Primers were synthesized by Metabion Company. The quantitative real-time polymerase chain reaction (qRT-PCR) reactions were performed using a Corbett machine, 72-well rotor using SYBR Green from ABI (Applied Biosystems, USA). All qRT-PCR reactions were run in duplicate, and we used the average threshold cycle (Ct) of 2 duplicates for further analysis. We used the housekeeping genes, *GAPDH* and *ß-Actin*, as they have the most homogenous expression level among the Royan hESC lines based on the NormFinder software analysis (data not shown). The geometric mean Ct of *GAPDH* and *ß-Actin* were calculated for each sample, and the Ct results from all 46 genes were normalized based on the mean housekeeping values.

### Directed neural differentiation of human embryonic stem cells

**Fig.1:**
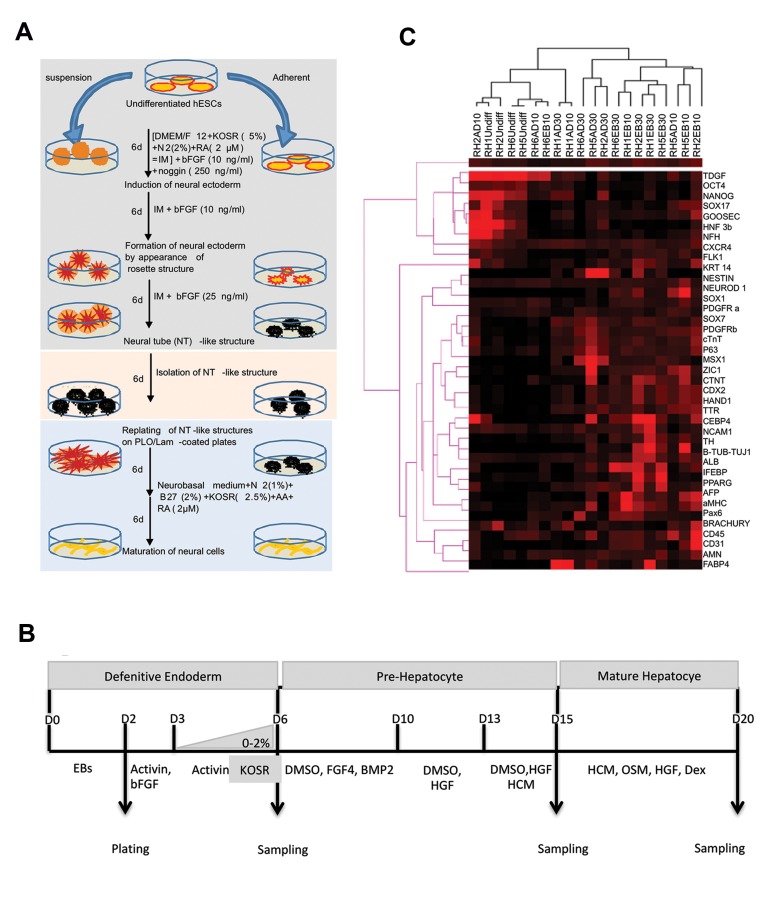
Differentiation protocols and clustering tree. **A.** The 3 steps of the neural induction and differentiation protocol, **B. **The 3 steps of the hepatic differentiation protocol, and** C.** Cluster of all samples based on expression levels of the 46 marker genes (vertical axis). hESCs; Human embryonic stem cells, N2; N2 supplement, RA; Retinoic acid, bFGF; Basic fibroblast growth factor, IM; Induction medium, AA; Amino acids, EB; Embryoid bodies, DMSO; Dimethyl sulfoxide, BMP; Bone morphogenetic protein, HGF; Hepatocyte growth factor, HCM; Hepatocyte culture medium, OSM; Oncostatin M, and Dex; Dexamethasone.

RH5 hESCs were differentiated to neuronal cells using 2 different protocols for confirmation of the spontaneous differentiation results. These protocols consisted of 3 main steps: i. Induction of hESC colonies toward neural ectoderm, ii. Differentiation toward neural tube formation, and iii. Neuron maturation stage (Fig .1A). Steps i and ii differed between the 2 protocols. In the first protocol, the cells were cultured in a suspension culture and they were grown in an adherent condition in the second protocol. The third step was identical for both protocols. Neural ectoderms were obtained by culturing hESCs in induction medium for 6 days, followed by 6 days in the same medium without Noggin. For induction of neural tube formation, the concentration of bFGF was increased to 25% and the cells were maintained in this medium for 6 days. For maturation, neural tubes (in adherent culture) and neuronal precursor cells (in suspension condition) were transferred to laminin/ poly-L-ornithine culture dishes and grown for 12-14 days in maturation medium. Samples were collected from all the 3 stages for both differentiation protocols.

### Directed hepatic differentiation of human embryonic stem cells

We used 2 hESC lines (RH2, RH6) at passages 25-35 to differentiate into a hepatic lineage according to the protocol of Basma et al. (37) with some modifications (Fig .1B). Briefly, EBs were generated by plating collagenase/dispase-passaged cells at a density of 1-5×104 cells/cm2 on bacterial petri dishes for 48 hours in DMEM/F12 supplemented with 20% KOSR, 1 mM nonessential amino acids, and 2 mM L-glutamine. Then, EBs were plated on Matrigel-coated plates in DMEM/F12 supplemented with Activin A (100 ng/ ml) for 6 days to induce definitive endoderm lineage. The day-6 cells were used as definitive endoderm for analysis. The concentration of KOSR was 0% for the first 48 hours, 0.2% for the second 24 hours, and 2.0% for the final 24 hours. Cells were then grown for 3 days in DMEM/F12 that contained 2.0% KOSR, 1 mM nonessential amino acids, 2 mM L-glutamine, 1% dimethyl sulfoxide, 10 ng/ml fibroblast growth factor 4 (FGF4, Royan Biotech, Iran), and 20 ng/ ml bone morphogenetic protein 2 (BMP2, Sigma, USA). The cells were allowed to grow in the same base media for an additional 4 days with 100 ng/mL hepatocyte growth factor (HGF, Sigma, USA) instead of FGF4 and BMP2. Next, they were cultured for 7-8 additional days in hepatocyte culture medium (HCM, Lonza, Swiss) that contained 2% KOSR, 1 mM nonessential amino acids, 2 mM L-glutamine, and 50 ng/ml HGF for the first 2 days as pre-hepatocyte cells at this step, followed by 5-6 days in maturation media that contained the same base media with 20 ng/ ml oncostatin M (OSM, Royan Biotech, Iran), 10 ng/ ml HGF, and 0.1 µM dexamethasone. 

### Statistical analysis

qRT-PCR results were converted to relative concentrations based on the standard curve method. Analysis of variances was performed on the readings from 60 samples and 48 different transcripts. We used the Statistical Analysis System (SAS) for 2-factor ANOVA by considering the hESC lines and the culture methods as 2 variable factors. P<0.01 were considered to be significant. SAS also provides a Duncan grouping chart for each gene in which samples are sorted based on that gene’s expression level. We used Eisen Lab and TreeView softwares for hierarchical clustering of the samples and genes.

## Results

### Gene expression profiles of the different hESC lines begin to diverge during differentiation

Clustering of the complete data set, as shown in Figure 1C, indicates that different hESC lines have similar expression profiles at the undifferentiated stage. Once the cells start to spontaneously differentiate, their gene expression profiles differ significantly due to their distinct intrinsic lineage propensities. As expected, most of the genes that were markers of the same cell type were clustered in close proximity in the clustering tree, which indicated reliability of the results. Pluripotency markers Oct4, Nanog, and TDGF were all clustered together, as were the endoderm markers HNF3b, CXCR4, and SOX17.

### CHD1 temporal expression pattern differed in comparison with pluripotency markers

**Fig.2:**
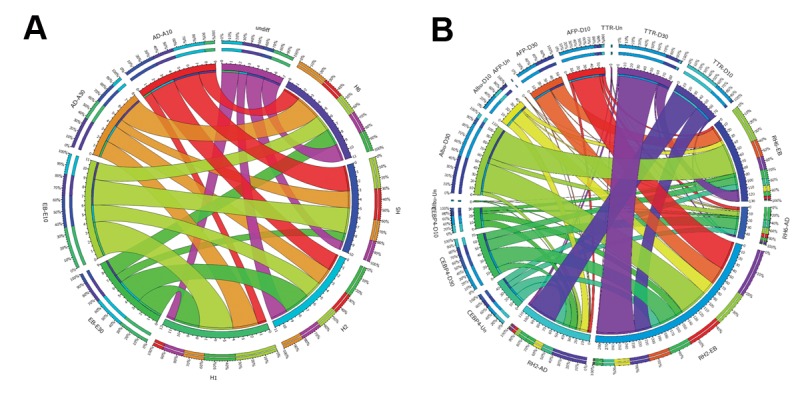
Expression comparison of spontaneous differentiation in different conditions. A. The expression level of CHD1 peaked at day 10 of spontaneous differentiation in both the suspension and adherent culture systems and B. Comparison of the expression levels of liver-specific markers between the Royan H2 (RH2) and RH6 lines during spontaneous differentiation in suspension [embryoid body (EB)] and adherent (AD) conditions.

CHD1, a chromatin remodeler known to be involved in formation and maintenance of the open chromatin state, showed interesting results. We assessed the expression levels of CHD1 in 4 hESC samples, and at days 10 and 30 after spontaneous differentiation. Figure 2A shows that the expression level of CHD1 increased upon differentiation and peaked at day 10, which was unexpected if it had a similar role in mouse ESCs and hESCs. A study on mouse ESCs showed that this protein highly expressed in stem cells and was responsible for the existence of a completely open chromatin in undifferentiated stem cells, and was required for the stemness property of mESCs.

### Expression levels of lineage-specific markers showed variations among different cell lines

**Fig.3:**
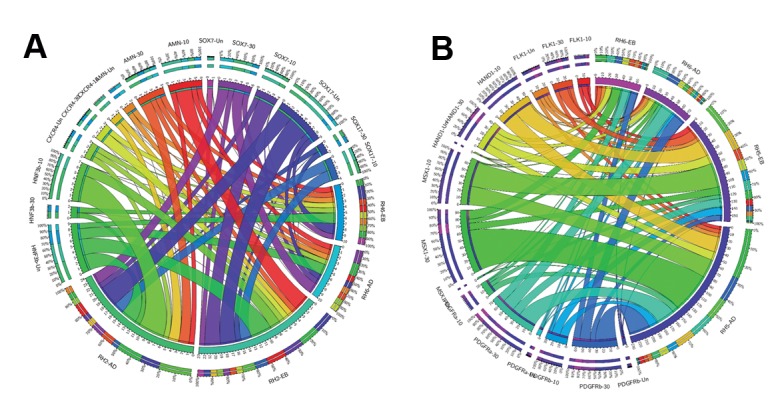
Expression comparison of endodermal and mesodermal markers between Royan embryonic stem cell (ESC) lines. A. Comparison of the expression levels of endodermal markers between the Royan H2 (RH2) and RH6 cell lines during spontaneous differentiation in suspension [embryoid body (EB)] and adherent (AD) conditions and B. Comparison of the expression levels of mesodermal markers between RH6 and RH5 cell lines during spontaneous differentiation in EB and AD conditions.

**Fig.4:**
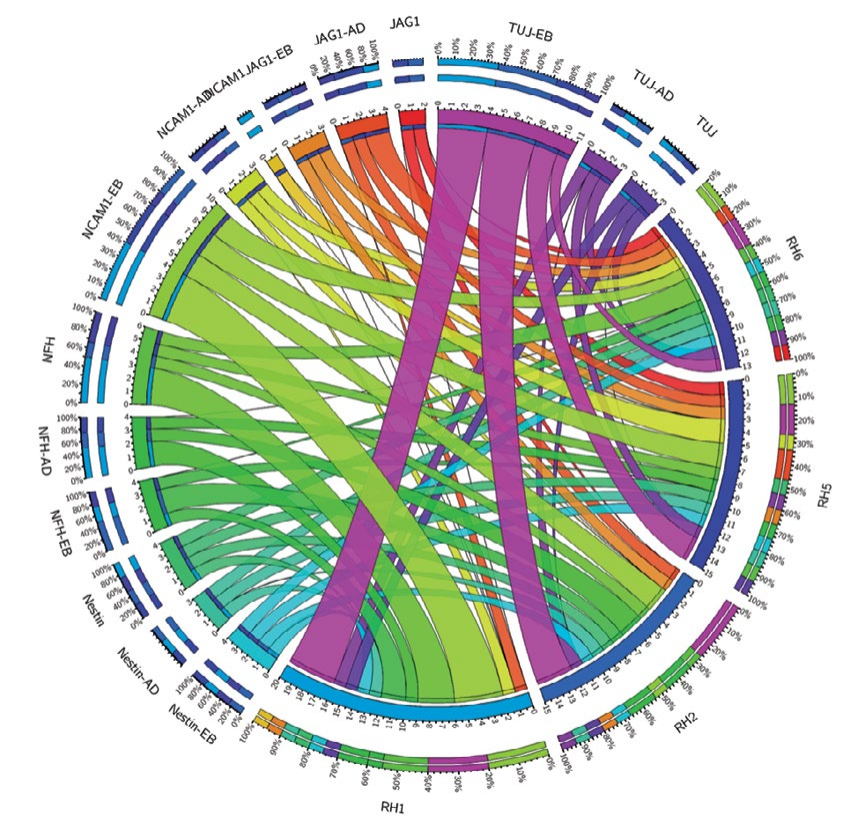
Relative expression levels of 3 neural markers in the 4 human embryonic stem cell (hESC) lines during spontaneous differentiation in suspension [embryoid body (EB)] or adherent (AD) culture systems.

**Fig.5:**
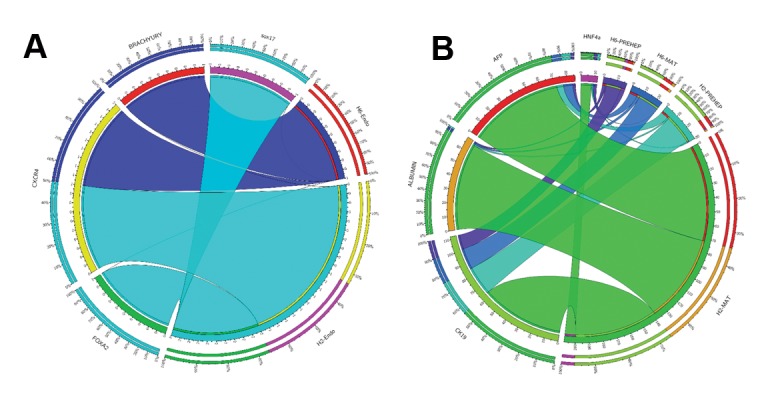
Relative expression levels comparison between RH2 and RH6 cell lines. A. four endodermal markers and B. four hepatocyte markers in directed differentiation of the Royan H2 (RH2) and RH6 cell lines in 3 stages: endoderm (ENDO), pre-hepatocyte (PREHEP), and mature hepatocyte.

A comparison of lineage-specificity among the 4 hESC lines in the current study was the first step for additional approval of the reliability of marker expression levels in spontaneous differentiation. The majority of the 46 examined markers showed significant differences in gene expression patterns among the 4 lines, which suggested divergent lineage specification. Based on spontaneous differentiation results, we selected 2 hESC lines for directed differentiation tests. The RH2 line showed the highest propensity to express endodermal (Fig .2B) and hepatocyte markers (Fig .3A). RH6 showed the lowest propensity to express endodermal (Fig .2B) and hepatocyte markers (Fig .3A). RH5 had the highest relative propensity to express the mesodermal (Fig .3B) and neural markers (Fig .4). We selected the RH2 and RH6 lines for further study on the comparison of directed differentiation toward hepatocytes under identical conditions. qRT-PCR results of directed differentiation towards endodermal and hepatocytes showed that RH2 had significantly higher efficiency to differentiate to endoderm and hepatocytes (Fig .5). There was a substantial distance between RH2 and RH6 based on the hierarchical clustering tree for hepatic markers (Fig .S1) (See Supplementary Online Information at www.celljournal.org). These results confirmed previous researches on determination of lineage specificity among different stem cell lines (29, 30). 

### Different cell lines showed differentiation potential in suspension and adherent cultures

**Fig.6:**
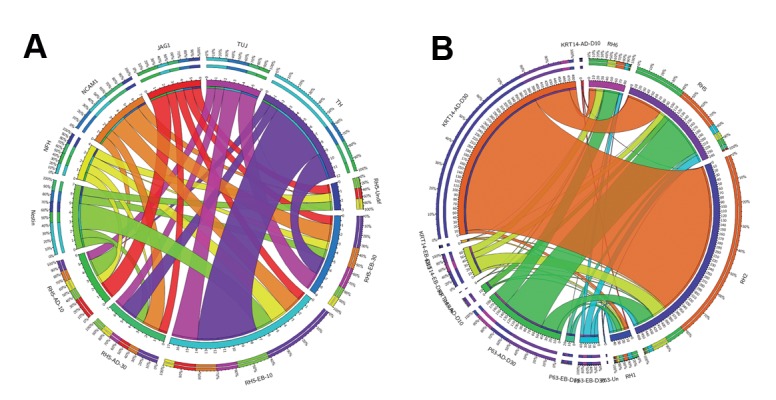
Expression comparison of neural and keratinocyte markers in adherent or suspension culture conditions. A. Relative expression levels of 6 neural markers in Royan H5 (RH5) during spontaneous differentiation under adherent or suspension culture conditions and B. Relative expression levels of keratinocyte markers, P63 and KRT14, under suspension [embryoid body (EB)] and adherent (AD) culture conditions in 4 human embryonic stem cells (hESC) lines (note the scale on the Y-axis).

Primary results confirmed the effectiveness of spontaneous differentiation in a comparison of the different hESC lines. In the second step, we compared EB versus the adherent culture methods using the same approach. Gene expression levels in suspension and adherent culture were compared for all lines. Interestingly, different lineage markers showed differential patterns in each condition. The ectodermal, neural, endodermal, hepatic, and endothelial markers had higher expression levels in suspension condition (Fig .6A), while the expression of skin-specific markers was higher in the adherent system (Fig .6B). 

### Directed neural differentiation of human embryonic stem cells

We confirmed the spontaneous differentiation results by direct differentiation of the RH5 cell line into neural cells according to the 2 induction protocols. The first protocol comprised the suspension culture stage, whereas the second protocol used the adherent culture during differentiation. Results of directed differentiation of stem cells to neurons confirmed spontaneous differentiation results, which showed that the suspension protocol was significantly more effective than the adherent culture.

Our results confirmed that the expression level of neural specific expressing markers like GFAP, PAX6, PDGFRa (as general neural stem cell markers) and b-Tublin (as general pre-mature neuronal marker) are higher in suspension (EB form) than adherent system in both direct and spontaneous differentiation systems, even in neuro-ectodermal specification or neural maturation stages. Although in both differentiation systems we have acceptable increase in neural lineage specific markers (Fig .S2) (See Supplementary Online Information at www. celljournal.org).

### Directed endodermal and hepatic differentiation of human embryonic stem cells

Spontaneous differentiation results showed that RH2 and RH6 had significant differences in expression of endoderm and hepatocyte markers. According to these results, RH2 differentiated into endodermal and hepatocyte cells with higher efficacy compared to RH6. Direct endodermal and hepatic differentiation confirmed that spontaneous differentiation could be a powerful tool to predict the propensity of hESC lines. Direct differentiation results verified findings that the RH2 cell line had significantly higher expression levels of endodermal and hepatic markers (Fig .5). According to these data, spontaneous differentiation analysis could be a reliable, rapid, and economical method for optimization of differentiation protocols.

## Discussion

hESCs have remarkable potential as cell sources for cell-based therapies. However, lack of knowledge in many aspects of hESC biology is a main barrier for introduction of stem cells in the clinic. In this study, we have focused on optimization of hESCs differentiation toward desired lineages, as one of the most important challenges in stem cell applications. Prior to using stem cells in the clinic, 2 major obstacles must be solved-differentiation efficiency and purity of differentiated cells. However, both must have the capability to produce purified cells at the maximum rate. Optimization of current direct differentiation protocols can overcome these problems. Here, we have investigated the effects of 2 parameters, cell line and dimension (2D vs. 3D). Both parameters had a significant influence on the final results. Although the effect of dimension has been extensively discussed, there are few reports that have compared different lineages in 2D versus 3D culture systems. More recently, the 3D culture systems have been developed to recapitulate human complicated organs (like nervous system) development and differentiation in in vitro system starting from human pluripotent stem cells (hPSCs) by organoid technology (cerebral organoid models). Although many studies have been conducted to reveal the mechanisms of 3D differentiation in higher organs studies but there are still many questions to be addressed (38). In the most suspension differentiation system, increases in expression levels of region specific neural genes are shown. That these changes in the expression level of specific genes are mostly described by mysterious cell-cell interaction and releasing neurotrophic factors from specific regions of EBs (spheroids), and 3D self-organization of ESCs in suspension culture system (39).

## Needed cell types dictate using suspension or adherent culture systems

We observed that the ectodermal, neural, endodermal, hepatic, and endothelial markers had higher expression levels in cells grown in the suspension condition, while skin-specific markers were expressed more in the adherent system. A possible explanation could be the similarity of the ex vivo environment to the natural extracellular environment for each cell or tissue type (40). Keratinocytes usually grow in a 2D layered condition in the body; hence these cells would prefer the adherent culture system. On the other hand, neural cells and hepatocytes grow in the 3D state in organs. Thus, they had more efficient differentiation in suspension culture. Cell-cell interactions and some signaling pathways might also be involved in the hESC response to the culture condition. Further studies would be needed to reveal the mechanisms that underlie lineage fate determination of hESCs during early differentiation stages of different culture methods. According to the current study results, some of the differentiation protocols could be improved by addition of a suspension step in the early stages of these protocols.

## Spontaneous differentiation has the potential to predict the behavior of hESC lines in direct differentiation

Gene expression profile analysis of hESCs during differentiation is a simple and reliable approach to predict their lineage propensities. This cost effective method could provide very useful data over a short period of time instead of cultivation of different hESC lines, differentiating them to all possible lineages, and comparing them. In the current study, we have used this invaluable tool to compare the expression levels of lineage-specific markers during spontaneous differentiation of hESC lines in suspension versus adherent culture systems. Although reports have shown this tool’s usefulness, we decided to confirm spontaneous differentiation results by direct differentiation of the RH5 cell line to neural cells. The direct differentiation data verified previous results and proved that this tool could be useful for optimization of hESC differentiation. According to the current results, the addition of a suspension culture step in differentiation of most lineages would be necessary. This new strategy may lead to the optimization of some common protocols used for neural differentiation of stem cells, and provide a standard platform for analysis of other types of differentiation protocols.

## Conclusion

Future studies, such as high-throughput analysis of the expression profiles on ESC lines during differentiation in adherent versus suspension culture conditions, are required for additional information in this area. Elucidation of the mechanisms that cause the early events of lineage specification under the 2 culture conditions is necessary. Commonly used differentiation protocols can be compared with respect to different environmental variables such as chemical and mechanical properties of the culture system to enhance the efficiency of stem cell differentiation toward a desired cell type, and further pave the way for stem cells to be used in a clinical setting. 

## Supplementary PDF


