# Supplementary material for: Gene Expression Patterns of Royan Human Embryonic Stem Cells Correlate with Their Propensity and Culture Systems
Source: Cell J. 2019 Jun 15;21(3):290–9. doi: 10.22074/cellj.2019.6128 (PMC6582416; doi:10.22074/cellj.2019.6128)
Supplement: Supplementary file 1 [file Cell-J-21-290-s01.pdf]

www.celljournal.org

Supplementary Information for

Gene Expression Patterns of Royan Human Embryonic Stem Cells Correlate with Their Propensity and Culture Systems

Hassan Rassouli, M.Sc.[1#], Mona Khalaj, M.Sc.[1#], Seyedeh-Nafiseh Hassani, Ph.D.[2], Shiva Nemati, Ph.D.[2], Ghasem Hosseini Salekdeh, Ph.D.[1, 3, 4*], Hossein Baharvand, Ph.D.[2, 5*]

1. Department of Molecular Systems Biology, Cell Science Research Center, Royan Institute for Stem Cell Biology and Technology, ACECR, Tehran, Iran
2. Department of Stem Cells and Developmental Biology, Cell Science Research Center, Royan Institute for Stem Cell Biology and Technology, ACECR, Tehran, Iran
3. Department of Systems Biology, Agricultural Biotechnology Research Institute of Iran, Agricultural Research, Education, and Extension Organization, Karaj, Iran
4. Department of Molecular Sciences, Macquarie University, Sydney, NSW, Australia
5. Department of Developmental Biology, University of Science and Culture, Tehran, Iran

#The first two authors equally contributed to this work.

**Corresponding Addresses: P.O.Box: 16635-148, Department of Molecular Systems Biology, Cell Science Research Center, Royan Institute for Stem Cell Biology and Technology, ACECR, Tehran, Iran*
P.O.Box: 16635-148, Department of Stem Cells and Developmental Biology, Cell Science Research Center, Royan Institute for Stem Cell Biology and Technology, ACECR, Tehran, Iran
Emails: salekdeh@royanInstitute.org, baharvand@royaninstitute.org

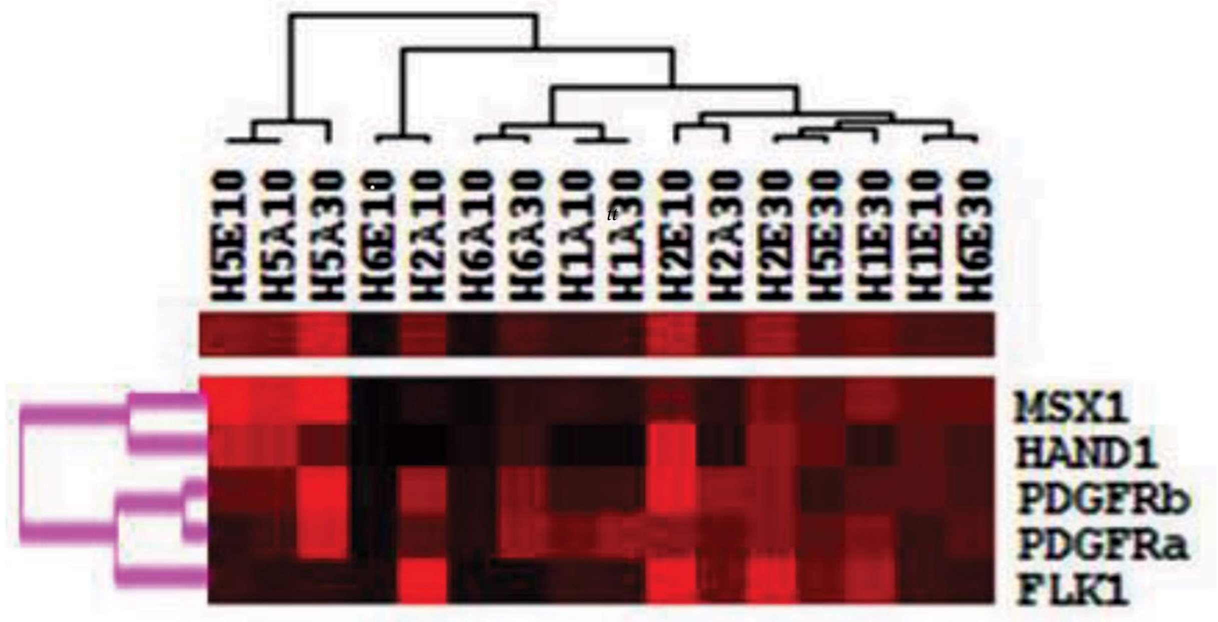

Fig.S1: Clustering tree of the samples based on the expression of liver-specific genes.

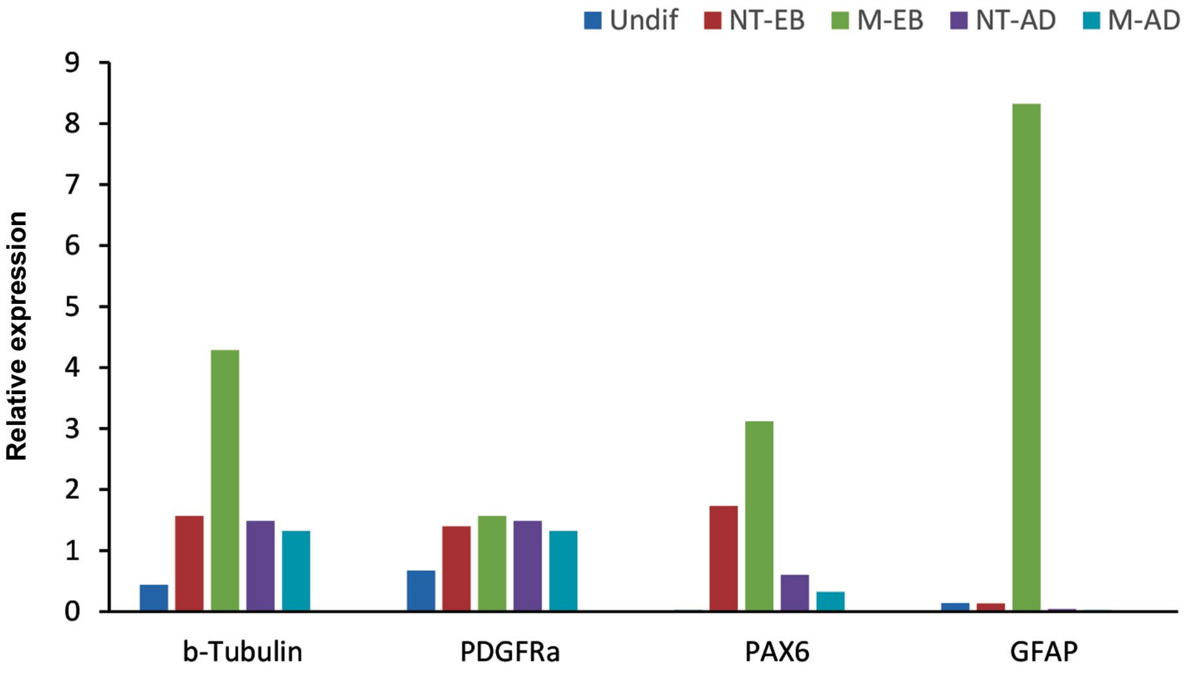

Fig.S2: Comparative expression levels of four neural markers in directed differentiation of RH5 to neural tubes (NT) and mature neurons (M) with the suspension (EB) or adherent (AD) protocols.

Table S1: Sequences of 48 primer used in current study

Gene name	Sequence primer (5´-3´)
Undifferentiated	
POU5F1	F: CTGGGTTGATCCTCGGACCT<br>R: CACAGAACTCATACGGCGGG
NANOG	F: AAAGAATCTTCACCTATGCC<br>R: GAAGGAAGAGGAGAGACAGT
TDGF	F: TCCTTCTACGGACGGAACTG<br>R: AGAAATGCCTGAGGAAAGCA
CDH1	F: GCTCTCCACTCTTACTTCCT<br>R: GTTTGGTCTGATGATGCG
Ectoderm	
NEUROD1	F: TCTCAGTTCTCAGGACGA<br>R: CTTCTTCCTCCTCTTCCAG
SOX1	F: CACAACTCGGAGATCAGCAA<br>R: GGTACTTGTAATCCGGGTGC
PAX6	F: GTCCATCTTTGCTTGGGAAA<br>R: TAGCCAGGTTGCGAAGAACT
Ecto- and mesoderm	
ZIC1	F: CTGGCTGTGGCAAGGTCTTC<br>R: CAGCCCTCAAACTCGCACTT
Mesoderm	
MSX1	F: CAAGCTGCCAGAAGATGC<br>R: GGTTCGTCTTGTGTTTGC
HAND1	F: AACCTCAGCCCTATCTCC<br>R: CTTTCATCTTCCTGCGTC
FLK1	F: TGATCGGAAATGACACTGGA<br>R: CACGACTCCATGTTGGTCAC
PDGFR-α	F: ACAGGTTGGTGTGGGTTCAT<br>R: CTGCATCTTCCAAAGCATCA
PDGFR-β	F: CCCTTATGTCGGAGCTGAAG<br>R: GCGGCAGTACTCAGTGATGA
Mesendoderm	
BRACHYURY	F: AATTGGTCCAGCCTTGGAAT<br>R: CGTTGCTCACAGACCACA
GOOSECOID	F: GAGGAGAAAGTGGAGGTCTG<br>R: CTCTGATGAGGACCGCTTCTG
Definitive and extraembryonic endoderm	
CXCR4	F: AACTTCAGTTTGTTGGCTGC<br>R: CATTTCCTCGGTGTAGTTATCTG
HNF4a	F: ATGACAATGAGTATGCCT ACCT<br>R: GGTCGTTGATGTAGTCCTCC

Table S1: Continued

Gene name	Sequence primer (5´-3´)
SOX17	F: CTCTGCCTCCTCCACGAA<br>R: CAGAATCCAGACCTGCACAA
HNF3β	F: GGAGCGGTGAAGATGGAA<br>R: TACGTGTTCATGCCGTTCAT
AMN	F: GACTCTGACCGCTTCTCCTG<br>R: CACTAGGCGGAAAGAAGACG
SOX7	F: ACGCCGAGCTCAGCAAGAT<br>R: TCCACGTACGGCCTCTTCTG
Neuron	
NFH	F: ACCTGCTCAATGTCAAGA AG<br>R: CAATCCGACACTCTTCACCT
JAGGED1	F: GATATACGGGATGATGGG AAC<br>R: GCAAGGGAACAAGGAAATCT
NCAM1	F: TCCCACCAACCATCATCTG<br>R: ACAGCGATAAGTGCCCTC
NESTIN	F: TCCAGGAACGGAAAATCAAG<br>R: GCCTCCTCATCCCCTACTTC
β-TUBULIN	F: CAGATGTTCGATGCCAAGAA<br>R: TGCTGTTCTTGCTCTGGATG
TH	F: CTGTGGCCTTTGAGGAGAAG<br>R: ATGGTGGATTTTGGCTTCAA
NCAM	F: TCC CAC CAA CCA TCA TCT G<br>R: ACA GCG ATA AGT GCC CTC
Skin	
P63	F: TTTCAGAGGCAATCCACACA<br>R: ATGCATGCAAATGAGCTCTG
KRT14	F: GACCATTGAGGACCTGAGGA<br>R: CATACTTGGTGCGGAAGTCA
Heart	
NKX2-5	F: CCCACGCCCTTCTCAGTCAA<br>R: GTAGGCCTCTGGCTTGAAGG
cTnT	F: ATGATGCATTTTGGGGGTTA<br>R: CAGCACCTTCCTCCTCTCAG
α-MHC	F: ACCCAAGTTCGACAAAATCG<br>R: TAAGGGTTGACGGTGACACA
GATA4	F: CCTGTCATCTCACTACGG<br>R: GCTGTTCCAAGAGTCCTG
Blood cells and endothelia	
CD45	F: TGTGATGCTTGTTCCCTTCA<br>R: ACTGGAGTGTGGAGCAGCTT
CD31	F: CCTGTCTTTCAGCCTTCAGC<br>R: CGCCTGTGAAATACCAACCT

Table S1: Continued

Gene name	Sequence primer (5´-3´)
Fat	
PPAR-γ	F: GCTGGCCTCCTTGATGAATA<br>R: TTGGGCTCCATAAAGTCACC
FABP4	F: CAGTGTGAATGGGGATGTGA<br>R: GGACACCCCCATCTAAGGTT
Pancreas	
PDX1	F: CCTTTCCCATGGATGAAGTC<br>R: GGAACTCCTTCTCCAGCTCTA
INSULIN	F: GGGGAACGAGGCTTCTTCTA<br>R: CACAATGCCACGCTTCTG
Liver	
TTR	F: CTGAGGAGGAATTTGTAG AAGG<br>R: GTGGTGGAATAGGAGTAGGG
CEBP4	F: TCTTTGAGTGTGGAGTGG<br>R: GGACAGACTTCTCTAGTTCG
ALBUMIN	F: CTTCCTGGGCATGTTTTTGT<br>R: TGGCATAGCATTCATGAGGA
AFP	F: AAATGCGTTTCTCGTTGCTT<br>R: GCCACAGGCCAATAGTTTGT
Intestine	
IFABP	F: TGCAGCTCATGACAATTTGA<br>R: CCCTGAGTTCAGTTCCGTCT
CDX2	F: GCAGAGCAAAGGAGAGGAAA<br>R: AAGGGCTCTGGGACACTTCT
Housekeeping	
β-ACTIN	F: CAATGTGGCCGAGGACTTTG<br>R: CATTCTCCTTAGAGAGAAGTGG
GAPDH	F: CTCATTTCCTGGTATGACAACGA<br>R: CTTCCTCTTGTGCTCTTGCT
